# Bilateral Lower Limb Sensorimotor Deficit Following General Anesthesia: A Case Report

**DOI:** 10.7759/cureus.93886

**Published:** 2025-10-05

**Authors:** Ahmed Bogari, Khlod K AlHarbi

**Affiliations:** 1 Anesthesiology and Intensive Care, King Fahad General Hospital, Jeddah, SAU; 2 Anesthesia, King Fahad General Hospital, Jeddah, SAU

**Keywords:** bilateral sensory loss, case report, general anesthesia, magnetic resonance imaging, postoperative neurological deficit

## Abstract

Bilateral lower-limb sensorimotor deficit after general anesthesia is rare but important. We describe a 36-year-old woman who developed acute bilateral lower-limb weakness and numbness immediately after elective supra-umbilical hernia repair under general anesthesia. Notably, she had experienced a similar transient episode following cesarean section anesthesia. She had a negative diabetic history, no baseline numbness in daily activities, and a preoperative exam confirming all motor and sensory reflexes intact. Past history was significant for antidepressant therapy. Intraoperatively, induction was smooth, the patient remained vitally stable, and positioning was smooth in and out of the operating room. MRI excluded cord infarction and compressive lesions but revealed degenerative lumbar changes with paraspinal edema. Conservative management with dexamethasone, baclofen, and celecoxib led to complete recovery within four days. This case highlights the importance of recognizing perioperative neurological complications.

## Introduction

General anesthesia is commonly employed in various surgical settings, and while its safety profile is generally high, it occasionally results in neurological issues that are both rare and consequential [1].

Bilateral lower-limb sensory loss following general anesthesia is a rare but clinically significant postoperative complication. It manifests as numbness, tingling, or loss of sensation in both legs, potentially leading to functional impairment and patient distress. While general anesthesia itself does not directly cause sensory deficits, factors such as prolonged surgical positioning, nerve compression, spinal cord ischemia, and postoperative inflammatory responses can contribute to this condition [2].

In this case report, we describe a perioperative sensorimotor deficit with sensory predominance and documented lower-limb weakness in an otherwise stable patient, aiming to highlight the importance of thorough perioperative evaluation, detailed history-taking, and vigilant postoperative follow-up in identifying and managing such rare events.

## Case presentation

A 36-year-old female patient with a history of depression, managed with antidepressant medication, and a known smoker, was admitted for elective hernia repair under general anesthesia. Her medical history was also notable for lumbar disc disease. She had a negative diabetic history and reported no numbness during her daily activities other than the incidents during anesthesia. Of particular relevance, she had experienced a prior episode of transient bilateral lower-limb sensory loss following a cesarean section under general anesthesia, which had been resolved spontaneously without a definitive diagnosis.

Anesthesia was induced with propofol 2 mg/kg, fentanyl 1 µg/kg, and rocuronium 0.9 mg/kg and maintained with sevoflurane 2% in combination with multimodal analgesia, including paracetamol and dexamethasone (8 mg). Extubation was uneventful and facilitated with sugammadex (200 mg). The patient subsequently received pethidine (25 mg) for postoperative shivering. However, upon emergence from anesthesia, she reported an inability to move her lower limbs and noted significant bilateral numbness. Additionally, subcutaneous edema in the lower back was noted, likely reflecting postoperative inflammatory changes.

Neurological assessment in the recovery unit revealed reduced motor strength (3/5) in both proximal and distal lower-limb muscles bilaterally. Sensory examination demonstrated diminished pinprick sensation from the feet to the mid-calf region. Preoperative examination had confirmed that all motor and sensory reflexes were intact. Muscle tone was not fully relaxed, but distraction testing indicated normal tone. Deep tendon reflexes were preserved at +2 throughout, while plantar responses were mute, and no clonus was elicited. Proprioception remained intact, and the straight leg raise test was positive bilaterally.

**Figure 1:**
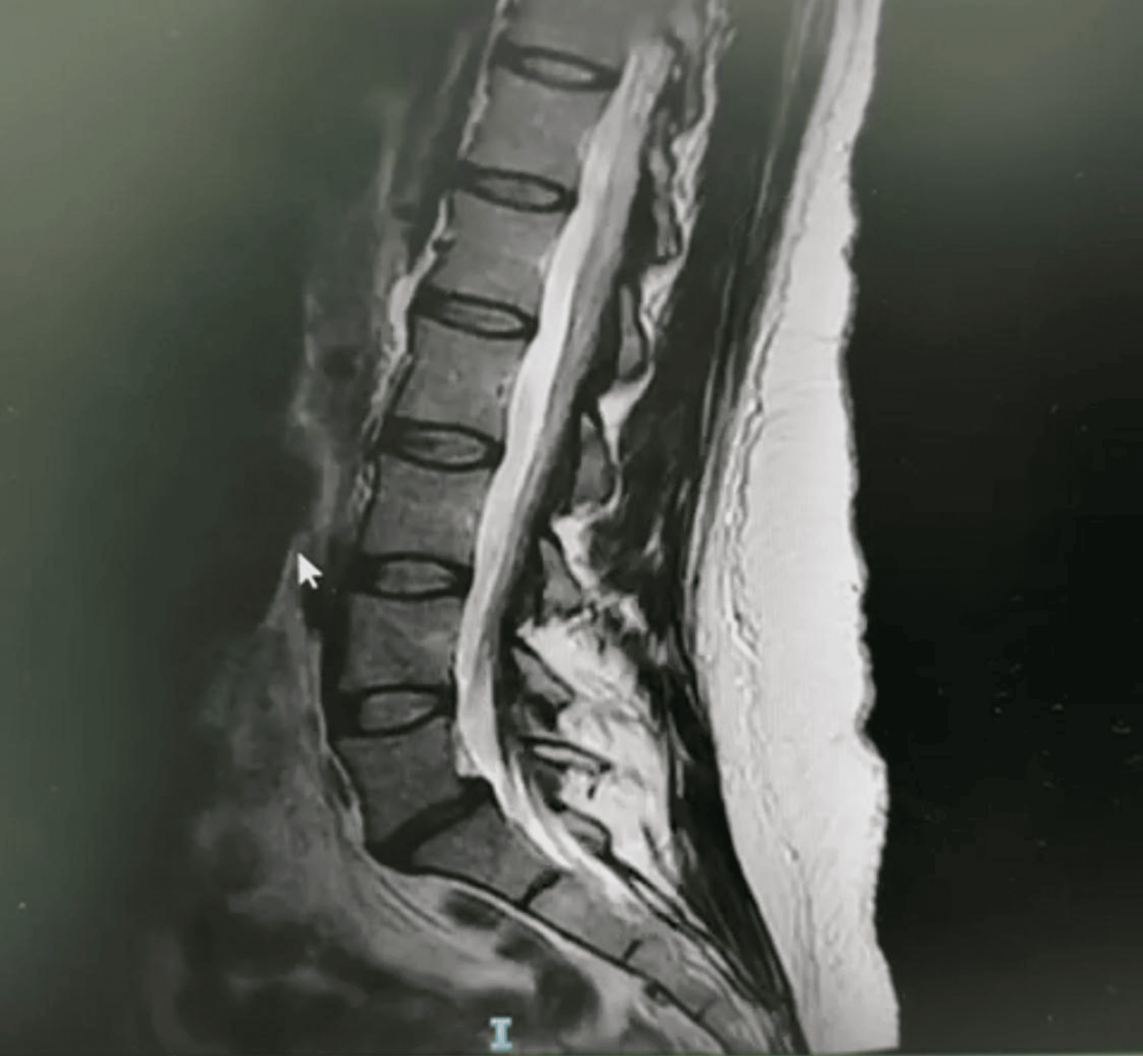
MRI findings showed no evidence of spinal cord infarction, acute ischemia, or compressive lesions

An urgent thoracic and lumbar spine MRI was performed to exclude a vascular insult or structural spinal pathology. MRI findings showed no evidence of spinal cord infarction, acute ischemia, or compressive lesions. The imaging did, however, reveal moderate lumbar spondylosis, L5-S1 disc disease with grade I anterolisthesis, mild bilateral foraminal narrowing, and fatty infiltration of the bilateral paraspinal muscles (Figure 1).

Intraoperative details were notable for smooth induction, stable vital signs throughout the operation, and appropriate positioning both inside and outside the operating room. Nerve conduction analysis was not performed.

The absence of an acute ischemic event suggested that the patient's neurological deficits were most likely attributable to underlying degenerative spinal pathology, potentially exacerbated by intraoperative positioning or transient inflammatory responses.

The patient was managed conservatively with baclofen (10 mg once daily (OD), celecoxib (200 mg OD), and dexamethasone (8 mg IV every eight hours for five days). Close neurological monitoring was maintained, and over four days, the patient demonstrated gradual recovery of motor function and resolution of sensory deficits. By the time of discharge, she had regained full power and returned to her usual functional state.

## Discussion

Given the abrupt onset of neurological deficits postoperatively, spinal cord ischemia was a significant initial concern. Ischemic spinal cord injury is rare but can result from intraoperative hypotension, vascular compromise, or embolic events, especially in patients with vascular risk factors such as smoking [3]. However, the MRI findings showed no evidence of infarction or ischemia, ruling out this possibility.

The MRI findings of L5-S1 disc disease with anterolisthesis and mild foraminal narrowing suggest chronic degenerative changes that may have predisposed the patient to transient neurological dysfunction. Prolonged intraoperative positioning (e.g., lithotomy or exaggerated Trendelenburg) may have led to nerve root irritation or transient compression, resulting in the observed motor and sensory deficits [4].

The noted postoperative subcutaneous edema in the lower back may have reflected an inflammatory response exacerbating preexisting nerve root irritation. The management of postoperative neurological deficits in this case was based on a multimodal approach targeting inflammation, neurogenic dysfunction, and supportive care.

Dexamethasone was used as part of multimodal analgesia and subsequently in treatment, suggesting a possible role of transient neurogenic inflammation in symptom development [5]. As a potent corticosteroid, dexamethasone exerts its effects by inhibiting pro-inflammatory cytokines such as IL-1, IL-6, and TNF-α, which are key mediators in neural inflammation and edema. It also reduces vascular permeability, thereby minimizing nerve root swelling and spinal cord edema. Furthermore, by suppressing leukocyte migration, dexamethasone prevents further inflammatory damage to affected neural structures [5].

The presence of postoperative subcutaneous edema in the lower back on MRI suggested an inflammatory component, making corticosteroid therapy a rational choice. Studies [6,7] have shown that corticosteroids significantly reduce neuropathic pain and improve functional recovery in patients with nerve root inflammation.

Baclofen was administered to address possible spasticity and neurogenic dysfunction. As a gamma-aminobutyric acid (GABA)-B receptor agonist, baclofen works by inhibiting excitatory neurotransmission, thereby reducing involuntary muscle contractions and spasticity. It also decreases alpha-motor neuron excitability, which helps prevent muscle stiffness and improve motor function. Given the patient’s initial lower-limb weakness and positive straight leg raise test, baclofen likely contributed to symptom relief by modulating spinal excitability. Baclofen improves motor recovery in cases of transient spinal cord dysfunction, highlighting its effectiveness in neurogenic recovery [8].

The administration of celecoxib, a selective cyclooxygenase-2 (COX-2) inhibitor, was aimed at reducing pain and inflammation associated with lumbar disc disease. Celecoxib works by inhibiting prostaglandin synthesis, alleviating inflammation while minimizing gastrointestinal side effects compared to nonselective nonsteroidal anti-inflammatory drugs (NSAIDs). Additionally, COX-2 inhibitors provide neuroprotective effects by preventing inflammatory cascades that could exacerbate nerve root compression. Given the patient’s preexisting lumbar spondylosis and foraminal narrowing, celecoxib likely contributed to symptom relief [9]. COX-2 inhibitors effectively reduce radicular pain in lumbar disc disease without negatively affecting neural function [10].

Supportive management, including close neurological monitoring and early mobilization, was crucial for recovery. Frequent neurological assessments ensured the timely detection of any worsening symptoms, allowing for appropriate interventions. Early mobilization helped prevent complications such as deep vein thrombosis (DVT) and muscle atrophy, which are common risks in patients with transient neurological deficits. Tazreean et al. [11] indicated that early mobilization in patients with postoperative neurological dysfunction enhances recovery and prevents secondary complications. The gradual improvement observed over four days without invasive interventions further supported a transient, reversible pathology rather than a permanent spinal cord injury.

## Conclusions

The development of a perioperative sensorimotor deficit with sensory predominance and documented lower-limb weakness after general anesthesia was most likely related to underlying lumbar disc disease and transient inflammatory or positional factors, rather than acute ischemia or structural compression. The patient’s complete recovery within four days under conservative management (dexamethasone, baclofen, and celecoxib) underscores the importance of early recognition, careful postoperative monitoring, and individualized treatment in similar presentations. Even in the absence of definitive radiological pathology, reversible neurological deficits may occur and require prompt clinical attention.
